# Effect of Agro-Industrial by Products Derived from Volatile Fatty Acids on Ruminant Feed In Vitro Digestibility

**DOI:** 10.3390/ani14162330

**Published:** 2024-08-12

**Authors:** Milad Parchami, Bengt-Ove Rustas, Mohammad J. Taherzadeh, Amir Mahboubi

**Affiliations:** 1 Swedish Centre for Resource Recovery, University of Borås, 501 90 Borås, Sweden; milad.parchami@hb.se (M.P.); mohammad.taherzadeh@hb.se (M.J.T.); 2 Department of Animal Nutrition and Management, Swedish University of Agricultural Sciences, P.O. Box 7024, 750 07 Uppsala, Sweden; bengt-ove.rustas@slu.se

**Keywords:** agro-food byproducts, acidogenic fermentation, membrane bioreactor, ruminant feed alternative, sustainability

## Abstract

**Simple Summary:**

The problem of food and agricultural waste, known as agro-food byproducts (AFB), is growing, and researchers are trying to find ways to turn this waste into something useful. One promising solution is acidogenic fermentation, a process that converts AFB into volatile fatty acids (VFAs) that can potentially be added to animal feed, providing energy and reducing reliance on traditional feed components like silage and concentrate. The main goal of this study was to determine if adding VFAs to ruminant (for example, cow) feed would affect digestion and fermentation in the rumen (the first stomach). Using a lab method called the Menke gas method, we tested how VFAs impact gas production, including methane. The results showed that VFAs can replace some of the energy usually provided by silage and concentrate without disrupting the fermentation process. Specifically, replacing 10% of the energy from silage and 20% from concentrate with VFAs in a mixed feed could reduce methane emissions while supplying enough energy for the animals. This approach benefits society by reducing waste from food and agriculture, lowering methane emissions from livestock, and producing more sustainable meat and dairy products.

**Abstract:**

The growing demand for sustainable ruminant feed alternatives has motivated the application of bioconversion approaches for the valorization of agro-food byproducts (AFB) into feed additives and supplements. The present study thoroughly investigated substituting volatile fatty acids (VFAs) obtained from acidogenic fermentation (AF) of AFB as an energy source in ruminant feed. Rumen in vitro digestibility assays were conducted utilizing the gas production method, wherein the VFAs obtained from AF of apple pomace and potato protein liquor was substituted with partial silage and concentrate energy at levels of 10%, 20%, and 30%. The results indicate that substituting 20% of the concentrate’s energy with VFA mixture significantly reduced methane production and had no adverse effect on the production and accumulation of VFAs in the simulated rumen media. Conversely, replacing 10% of the silage energy with VFAs led to a decrease in methane production and further enhanced the production of VFAs. Readily digestible VFAs in ruminant feed have the potential to enhance energy availability and sustainability in ruminant farming practices, aligning with the principles of circular economy and waste valorization.

## 1. Introduction

Agro-food byproducts (AFB) are the residual organic material generated during the production and processing of food and feed products. The AFB comprise a wide range of organic constituents, such as crop residues, discarded fruit and vegetable peels, and animal-derived materials [1]**,** leading to a substantial global waste output [2]. In 2011, the European Union (EU) generated a cumulative amount of 129 million metric tons of AFB. Each of these side streams is organic and can be efficiently utilized [3]. Agro-food side streams are typically regarded as having minimal value and are commonly utilized directly in animal feeding without processing [4]; incinerated; deposited in landfills; or, in more dire circumstances, disposed of in an unregulated manner [5]**,** resulting in significant environmental challenges and health concerns [6]. Researchers are now studying the viability of scaling up different processes such as pyrolysis, fermentation, and hydrothermal liquification to convert agricultural byproducts into valuable products such as biofuel, biochar, and biomaterials [7]. Furthermore, utilizing microorganisms that produce advantageous chemical compounds can function as an environmentally friendly alternative. A prime example of offering a sustainable solution to achieve this objective is acidogenic fermentation (AF). Acidogenic fermentation is a complex and multi-step process in which a consortium of anaerobic microorganisms converts organic materials into volatile fatty acids (VFAs) such as acetate, propionate, and butyrate. This technique offers the potential to convert agricultural byproducts, including olive oil mill effluent (yielding 371 g VFAs/kg dry matter), cheese whey, gelatin-rich proteinaceous wastewater, and sweet sorghum stalks (yielding 252 g VFAs/kg dry matter) [8], into high-value VFAs. These VFAs can serve as precursors for biofuels and platform chemicals [9,10].

One of potential uses of the VFAs is in ruminant nutrition as a feed ingredient [11]. Ruminants possess a distinctive digestive system in their gastrointestinal tract, which allows for the natural production of VFAs through anaerobic digestion of the ingested feed [12]. Ruminal VFAs play a pivotal role in providing energy to the post-absorptive ruminant metabolism [13]. Acetic acid serves as a primary substrate for fatty acid synthesis, propionic acid contributes to gluconeogenesis, and butyric acid is a direct energy source for rumen epithelial cells [11]. The effects of fossil-derived VFA salts, such as sodium acetate (NaAc), sodium butyrate (NaBu), and calcium propionate (CaPr), as additives in ruminant feed, have been extensively researched since the late 1950s. These studies have demonstrated that these salts have an impact on several physiological and health-related functions, as well as productivity [11].

The incorporation of biobased VFAs into the ruminant ration raises a thought-provoking question regarding the impact of supplementary VFAs on the digestibility of the feed. Intentional addition of VFAs to the feedstuffs such as silage and concentrate brings a new aspect to conventional feeding methods, which might possibly influence both the nutritional composition of the diet and the effectiveness of ruminal fermentation. Exploring the interplay between externally administered VFAs and the VFAs produced internally during ruminal digestion is an important area to investigate. One line of exploration to pursue is understanding how this supplementation affects the feed’s overall digestibility, as demonstrated by the production of biogas and the amount of VFAs in the rumen.

To test the hypothesis that the supplementary agro-food-byproduct-derived VFAs will not hamper the rumen fermentative activity, we conducted a rumen in vitro experiment using the Menke gas method [14]. This study systematically evaluates the impact of different levels (10–30% on energy basis) of VFAs replacement in silage and concentrate on ruminal gas production (mainly methane production rates and volumes) during ruminal fermentation in a controlled setting. Gas production was used as an indirect method to assess the digestibility of feed in a laboratory setting. Increased gas production indicates enhanced microbial degradation of the feed, implying greater digestibility for ruminants. Scientists utilize gas volume and incubation time to estimate the degree (organic matter digestibility) and rate of feed degradation by microorganisms. The objective is to evaluate the capacity of the VFA mixture as a feed component to augment energy content and partially substitute conventional energy sources.

## 2. Materials and Methods

### 2.1. Characterization of Feed Ingredients and the VFA Mixture

The VFA mixture used in this study was previously produced through the acidogenic fermentation of a mixture of apple pomace and potato protein liquid in a submerged microfiltration anaerobic membrane bioreactor [15]. The experiment was designed in an isoenergic scope. Hence, a percentage of feed energy was replaced by a volume of VFA mixture containing the same energy content. Silage and concentrate (con) were selected as the feed ingredients. Silage was collected from Husshållningssälskapet Sjuhärad (Länghem, Sweden). Protein concentrate was provided from a Grund balans, Svenska Foder AB (Lidköping, Sweden), and was dried and ground to pass a 2 mm screen. Silage consisted of hay silage, and concentrate consisted of wheat, corn, and rapeseed meal. The chemical compositions and energy content of the feed ingredients and VFA mixture are presented in Table 1. Each gram of acetate, propionate, and butyrate can provide 14.65, 20.93, and 25.12 kilojoules of energy, respectively [16].

### 2.2. In Vitro Experiment Design

The total energy content of the VFA mixture was estimated by considering the distribution of each acid and their respective calorific values [17]. However, the energy contribution from other components, such as ammonium, was not considered, since it is not typically considered as an energy carrier. This study included two independent experiments. Each experiment had three treatments to replace 10%, 20%, and 30% of feed gross energy with a VFA mixture solution (Table 2). About 400 mg hay silage and concentrate were used as a substrate in each condition, and based on the level of replaced energy, different amounts of ingredients were replaced with different volumes of VFA mixture.

Each treatment group had 10%, 20%, or 30% of its feed’s gross energy replaced with a specific VFA mixture solution. To achieve this, corresponding amounts of the starting feed were substituted with calculated volumes of the VFA mixture (based on energy content). For instance, to replace 10% of energy, 731.6 J of silage and 806.3 J concentrate must be replaced with 1.9 and 2.1 mL of VFA mixture, respectively. Therefore, these groups are denoted as the “silage1/10%VFAs” group and “Con1/10%VFAs” respectively. Each treatment group had a corresponding control group. Groups silage1, silage2, silage3, Con1, Con2, and Con3 served as the controls for treatment groups. The control group’s pH was adjusted to match the corresponding treatment group, ensuring a similar starting point for fermentation. All conditions were conducted in triplicate. Details on specific VFA mixture volumes, replaced feed amounts per treatment, and pH adjustments can be found in Table 2.

Rumen fluid was provided from fistulated cows by the Swedish University of Agricultural Sciences (SLU) (Lövsta, Sweden). The procedure was conducted according to the ethical approval held by Uppsala Animal Experiment Ethics Board, Sweden (diary no 5.8.8-11182/2019). The rumen fluid was sparged by CO_2_ and sealed with a one-way valve to be maintained under anaerobic conditions at 39 °C. The 120 mL serum bottles were used as fermentation vessels containing 20 mL of rumen fluid and 40 mL of the medium mixture. The medium mixture was prepared based on the Menke gas method protocol [18], consisting of (added in order) 400 mL H_2_O, 0.1 mL solution A (13.2 g CaCl_2_.2H_2_O, 10 g MnCl_4_. 4H_2_O, 1 g CoCl_2_. 6H_2_O, 8 g FeCl_3_, 6H_2_O, and made up to 100 mL with H_2_O), 200 mL solution B (39 g NaHCO_3_/L H_2_O), 200 mL solution C (5.7 g Na_2_HPO_4_, 6.2 g KH_2_PO_4_, 0.6 g MgSO_4_.7H_2_O, and made up to 1000 mL with H_2_O), 1 mL resazurine (0.1%, *w*/*v*), and 40 mL reduction solution (95 mL H_2_O, 4 mL l M NaOH, and 625 mg Na_2_S.9H_2_O). The mixture was kept under CO_2_ in a water bath at 39 °C and stirred by a magnetic stirrer prior to the test for 45 min. Different amounts of feed and VFA mixture were added to serum bottles containing 60 mL of rumen medium mixture, and the pH values of conditions in each trial were set at the pH of the mixture of feed and VFAs. This study consisted of two distinct sets of experiments, with each experiment comprising 21 bottles. To adjust the pH, an acid solution of 0.1 M HCl and a base solution of 0.1 M NaOH were used.

### 2.3. Analytical Method

Total Kjeldahl nitrogen (TKN) was examined according to the standard method presented by Parchami, Ferreira [19], and the nitrogen-to-protein conversion factor of 6.25 was used for converting the total nitrogen to protein [20]. The starch content of ingredients was measured using a starch kit (total starch kit K-TSHK, Megazyme, Bray, Ireland) [21]. Fiber analyzer A200 (ANKOM Technology, New York, NY, USA) was used for crude fiber (CF), neutral detergent fiber (NDF), and acid detergent fiber (ADF) determination following the protocols outlined in Ankom’s methodologies [22]. Moisture, volatile solid, and ash contents were analyzed using the standard method by Eaton, Clesceri [23]. Bomb Calorimeter, IKA 2000 (IKA-Werke GmbH and Co. KG, Staufen im Breisgau, Germany), was used to estimate the energy value of the concentrate and silage [24]. The analysis of ammonium nitrogen (NH_4_^+^–N) was performed using the Ammonium 100 test kit (Nanocolor, MACHEREY-NAGEL GmbH and Co. KG, Düren, Germany). NH_4_^+^–N concentrations were measured using the Nanocolor 500D Photometer (MACHEREY-NAGEL GmbH and Co. KG, Düren, Germany). By using gas chromatography (GC) (Clarus 590; Perkin-Elmer, Norwalk, CT, USA) benefitting from a packed column (CarboxenTM 1000, 6 × 1.8 OD, 60/80 mesh, Supelco, Shelton, CT, USA) and a thermal conductivity detector (TCD), the volume and composition of gas (CH_4_, H_2_, and CO_2_) was analyzed. The injection temperature for the GC-TCD was set to 200 °C, and the carrier gas was nitrogen at a flow rate of 30 mL/min at 75 °C.

VFA amount and distribution were determined using GC (Clarus 590; Perkin-Elmer, Norwalk, CT, USA) coupled to a capillary column (Elite-WAX ETR, 30 m × 0.32 mm × 1.00 m, Perkin-Elmer, Shelton, CT, USA) and flame ionized detector (FID). In the GC-FID condition, injection and detection temperatures were 250 and 300 degrees Celsius, respectively. The carrier gas was nitrogen with a flow rate of 2 mL/min and a pressure of 20 psi. VFA sample preparation and analysis was performed according to Parchami, Rustas [25]. The liquid samples were mixed with a solution of acids consisting of 25% (*v*/*v*) formic acid and 25% (*v*/*v*) ortho-phosphoric acid in a 1:3 ratio. The resulting mixture was then subjected to centrifugation at a speed of 10,000× *g* for a duration of 5 min. Subsequently, the liquid portion was passed through a 0.2 µm syringe filter to eliminate any solid particles that had not dissolved. An internal standard was employed using butanol at a concentration of 1 g/L.

### 2.4. Statistical Analysis

All experiments were conducted in triplicate. Statistical analysis was performed using MINITAB^®^ version 21 (Minitab Ltd., Coventry, UK). To assess the effect of VFA replacement level (0%, 10%, 20%, and 30%) on gas production (methane and CO_2_) and VFA production of the same samples measured at different points in time (hours 4, 8, 12, 24, 32, 48, 56, and 72), two-way ANOVA with repeated measures was conducted for the silage and concentrate experiments. Tukey’s honest significant difference (HSD) pair comparison test was used to determine which specific VFA replacement levels differed from each other in terms of gas and VFA production at that time point within each experiment (silage or concentrate).

## 3. Results

This study thoroughly examined the fermentation process of silage and concentrate, both alone and in combination with a solution of VFAs. The primary aim of this study was to evaluate the generated gases and VFA production during ruminal fermentation. The gas composition was studied at different time intervals to assess the influence of VFA supplementation on the microbial activity in the rumen and as an indicator of feed digestibility. Furthermore, the overall production of VFAs and the distribution of each acid were determined. This analysis aimed to establish a correlation between the generated VFAs, biogas, and the mixture of supplied VFAs.

### 3.1. Gas Production

#### 3.1.1. Gas Production in the Concentrate Energy Replacement Experiment

**Figure 1:**
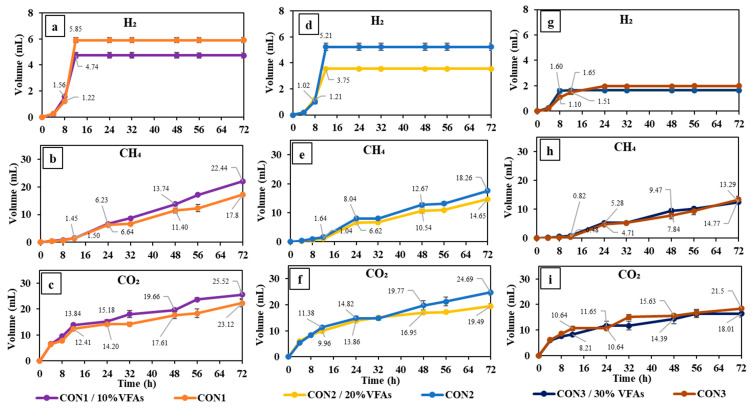
Cumulative gas production by incubation of concentrate and 10%VFA (**a**–**c**), 20%VFA (**d**–**f**), and 30%VFA (**g**–**i**) energy replacement.

The process of gas production through the fermentation of concentrate and a mixture consisting of concentrate and VFAs is illustrated in Figure 1. In addition, the comparison between variations and responses is given in Table 3. During the 72 h fermentation period, it was observed that Con1 and Con2 exhibited the highest levels of hydrogen (H_2_) production, measuring 5.9 mL and 5.2 mL, respectively. Both experimental settings exhibited the initiation of H_2_ production at hour 4, followed by a rapid increase in production until hour 12, reaching the maximum level. This maximum level was maintained until the conclusion of the trials. The utilization of various amounts of VFA mixture (10% and 20%) in conjunction with concentrate yielded similar outcomes in terms of enhancing H_2_ production. Thus, the replacement of 10% concentrate energy with a mixture of VFAs (Con1/10%VFAs) resulted in the highest amount of H_2_ compared to all other combinations of concentrate and varying degrees of VFA mixture, with a yield of around 4.7 mL. The experimental condition involving the combination of concentrate and 30%VFAs (Con3/30%VFAs) exhibited distinct characteristics in comparison to the preceding conditions. The production of H_2_ by Con3/30%VFAs was found to be comparable to that of Con3, with a cumulative volume of 1.7 mL at hour 8, and remained steady until hour 72. Replacing 10% of concentrate energy with VFA mixture led to the maximum methane (CH_4_) production when compared to higher levels of VFA replacement. The experimental group (Con1/10%VFAs) demonstrated a methane production of 22.4 mL during the 72 h fermentation period, which was significantly higher than the methane production of the relevant control group (Con1) at 17.8 mL (*p* = 0.01). The substitution of 20% energy with VFAs resulted in a notable reduction in methane generation compared to the control group, and this difference was statistically significant (*p* = 0.01). The replacement of 20% and 30% energy with VFA mixture led to the absence of methane production from hour 24 to 32, followed by a subsequent significant increase in methane production until hour 48 (*p* = 0.01). The lag times observed for the synthesis of hydrogen and methane were consistently 4 and 12 h across all experimental conditions, respectively. However, it is worth noting that no lag time was observed to produce carbon dioxide. During the period spanning from hour 0 to hour 72, Con1/10%VFAs exhibited the highest CO_2_ production, with Con2 following closely behind, producing 25.5 mL and 23.1 mL, respectively. Increasing the level of VFA replacement to 30% caused a drastic decrease in CO_2_ production. Con3/30%VFAs produced the lowest amount (18 mL) for 72 h fermentation.

#### 3.1.2. Gas Production in the Silage Energy Replacement Experiment

**Figure 2:**
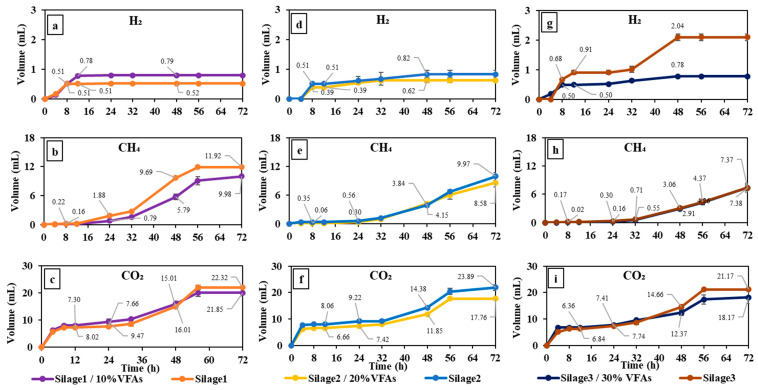
Cumulative gas production by incubation of silage and 10%VFA (**a**–**c**), 20%VFA (**d**–**f**), and 30%VFA (**g**–**i**) energy replacement.

The experimental procedure involved incubating silage and the VFA mixture in the rumen buffer media, resulting in the production of hydrogen, methane, and CO_2_. These findings are depicted in Figure 2. In addition, the comparison between variations and responses is given in Table 4. The substitution of 10% of the energy content in silage with the VFA mixture resulted in the production of 0.8 mL of hydrogen (as shown in Figure 2a). The level of H_2_ production in silage1/10%VFAs was found to be significantly higher (*p* = 0.03) when compared to the hydrogen production of silage without VFAs (silage1). The mechanism in which the increased levels of energy replacement occurred exhibited variation. The introduction of the VFA mixture as a replacement for 20% of the energy derived from silage (silage2/20%VFAs) resulted in a decrease in hydrogen production compared to the control group. This drop in hydrogen production was particularly pronounced when 30% of the silage energy was replaced (*p* = 0.03). For 72 h fermentation, silage3 and silage3/30%VFAs produced 2 and 0.8 mL H_2,_ respectively.

Silage1 produced the highest amount of CH_4_ among all conditions, which was 11.8 mL and followed by silage1+10%VFAs with 10 mL production (Figure 2b). Replacing higher energy levels (20 and 30%) with VFA mixture led to a lower methane production, wherein the combination of silage2/20%VFAs and silage3/30%VFAs produced 9.4 mL and 7.4 mL CH_4,_ respectively. However, the differences were not significant compared to their respective control groups (*p* = 0.08). Like CH_4_, silage1 exhibited higher CO_2_ production than silage1/10%VFAs (*p* = 0.02). CO_2_ production levels in the conditions of silage1 and silage1/10%VFAs were 22.3 and 20.1 mL, respectively (Figure 2f). Silage2 yielded a slightly higher volume than silage1, which was approximately 23.9 mL. However, higher VFA level replacement led to lower CO_2_ production. Replacing 20 and 30% VFAs produced 17.7 and 18.1 mL, respectively (Figure 2f,i).

### 3.2. Volatile Fatty Acid Production

#### 3.2.1. VFA Production in the Concentrate Energy Replacement Experiment

**Figure 3:**
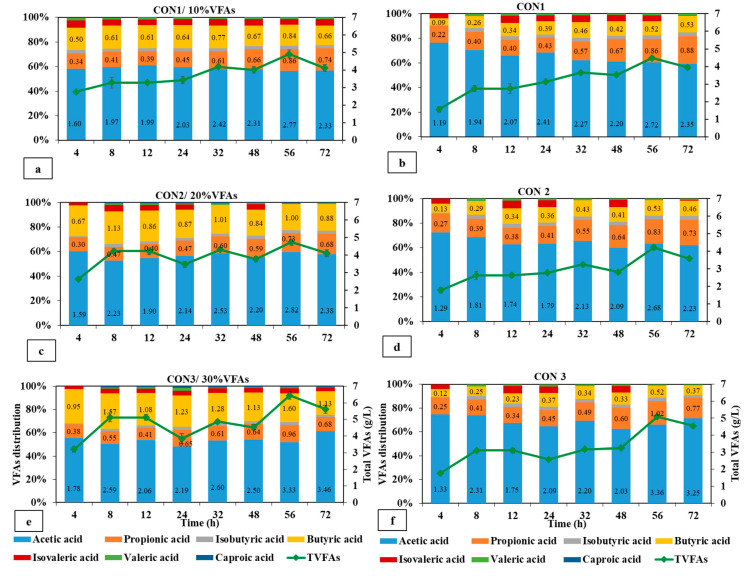
VFA production by replacing (**a**) 10%, (**c**) 20%, and (**e**) 30% concentrate energy with VFA mixture.

Figure 3 illustrates the production of VFAs through the process of fermentation using concentrate, with and without the addition of the VFA mixture. Replacing 10% and 20% of the concentrate energy with VFA mixture resulted in an increase in VFA production by hour 8. Con1/10%VFAs and Con1 exhibited similar trends throughout the whole period. Total VFAs reached their maximum at hour 56, with a value of 4.9 g/L in Con1/10%VFAs, and 4.5 g/L in Con1 and this level was maintained until hour 72 (Figure 3a,b) (*p* = 0.06). The total production of VFAs was determined by subtracting the initial VFA concentration at hour 4 from the final VFA concentration at hour 72. In this context, as can be seen in Table 3, Con1 exhibited a greater overall generation of VFAs at a level of 2.8 g/L, in contrast to Con1/10%VFAs, which yielded a lower amount of 1.6 g/L. While the level of total VFAs in Con2/20%VFAs was equivalent to that of Con1/10%VFAs at hour 4, Con2/20%VFAs showed a superior production rate compared to Con1/10%VFAs (*p* = 0.02). It is noteworthy to mention that elevating the energy replacement level by VFAs to 20% and 30% will intensify the fluctuations in VFA production over 72 h. According to the data presented in Figure 3e, the production of VFAs by Con3/30%VFAs showed a significant increase from 3.2 g/L at hour 4 to 5.1 g/L at hour 8 (*p* = 0.02). Subsequently, the production declined to 3.8 g/L at hour 24, and this pattern of fluctuations persisted until the conclusion of the experiment. While the fluctuations were less prominent in Con3, it was shown to create a lower level of VFAs compared to Con3/30%VFAs. The substitution of higher energy levels of concentrate with a VFA mixture at levels of 20% and 30% resulted in an increase in the production of VFAs and the distribution of acids. According to Table 3, the production of acetic acid and butyric acid was significantly higher in the Con3/30%VFA compared to the Con1/10%VFA condition (*p* = 0.01). Nevertheless, the experimental groups exhibited a similar level of acetic acid productivity compared to their respective control groups, but the butyric level was significantly higher in treatment groups Con3/30%VFAs and Con1/10%VFAs.

#### 3.2.2. VFA Production in the Silage Energy Replacement Experiment

**Figure 4:**
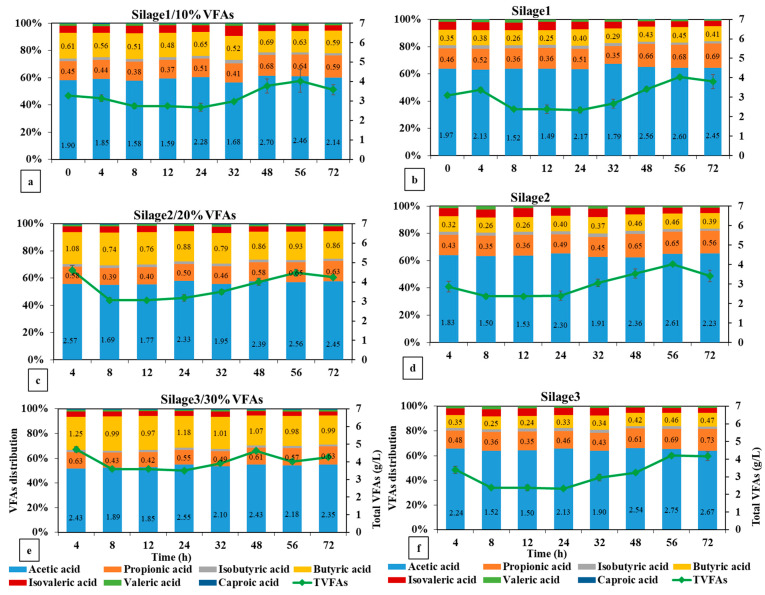
VFA production by replacing (**a**) 10%, (**c**) 20%, and (**e**) 30% silage energy with VFA mixture.

The substitution of energy in various feed constituents with a combination of VFAs will elicit distinct responses in terms of VFA production. Figure 4 illustrates the generation of VFAs by silage with and without the addition of the VFA mixture. The addition of VFA mixture to silage, by 10% replacement in energy content, led to higher concentrations of acetic and butyric acids compared to silage1 (*p* = 0.01). Specifically, the acetic acid concentration reached 2.8 g/L, while the butyric acid concentration reached 0.7 g/L. Consequently, the overall production of VFAs during the 72 h fermentation period was nearly twice as high as the quantity generated in the control group (silage1). Nevertheless, the addition of the VFA mixture at higher levels of silage energy (20% and 30%) did not change the production level of acetic and propionic acid as compared to their control groups. It is important to point out that the introduction of higher amounts of VFAs resulted in a noticeable downward trend in the total VFA production. While 10% energy replacement resulted in a more favorable increase in VFA synthesis, it was also accompanied by a lower methane output compared to silage1. In contrast, the experimental groups that received higher amounts of energy replacement with VFAs (20%, and 30%) had methane production rates comparable to those observed in their respective control groups.

Replacing a fraction of silage and concentrate with a VFA mixture resulted in distinct patterns in the VFA synthesis. The replacement of 10% energy in silage had a more favorable impact on VFA production compared to an equivalent replacement in concentrate. Silage1+10%VFAs generated 1.5 g/L of total VFAs, producing more butyric acid than the control group (*p* = 0.03). The experimental group for concentrate, which received 10%VFAs, showed a decrease in VFA production, as compared to the control group (Table 3). On the other hand, the introduction of silage energy replacement at levels of 20% and 30% VFAs resulted in a detrimental impact on VFA production over the entire duration of fermentation. Both experimental settings exhibited negligible formation of VFAs, but the control groups significantly yielded VFAs at levels of 1.2 and 1.4 g/L, respectively (*p* = 0.02). Nevertheless, when concentrate was supplemented with VFA solution, the energy replacement levels (20 and 30%) yielded higher VFA production. Con2+20%VFAs yielded a higher level of VFAs at 2.85 g/L compared to the 2.5 g/L obtained by the control group. Moreover, the addition of 30% of VFAs resulted in the production of VFAs that were comparable to those obtained from Con3.

## 4. Discussion

According to the findings presented in Figure 2, replacing 10% of the silage energy with VFAs had a notable impact on reducing CH_4_ production when compared to the control group (*p* = 0.03). However, this substitution led to an increase in the generation of H_2_, while it had no effect on reducing CO_2_ production. The hydrogenase enzyme, responsible for the conversion of protons into H_2_, particularly found in rumen protozoa and fungi, is hindered by CO_2_ concentrations, resulting in a shift of the fermentation process towards processes that use hydrogen, such as the generation of butyrate [26]. As can be seen in Table 3, the yield of butyric acid was notably higher in the group with 10% silage energy replacement, in comparison to its respective control group (*p* = 0.02).

It is worth mentioning that the levels of acetate and propionate exhibited consistency between the control groups, as well as VFA-supplemented groups. This indicates that the rumen microbial population efficiently regulated the utilization of externally provided VFAs in conjunction with those obtained from silage [27]. The stability highlights the resilience and flexibility of the rumen ecosystem. Hence, it can be asserted that there is the ability of rumen microbes to maintain stable level of acetate and propionate in response to dietary modifications, such as supplementation with VFAs, serving as evidence of their metabolic adaptability [28]. In the context of complex microbial communities, individual species possess the ability to adapt their metabolic pathways to maximize the utilization of substrates while simultaneously preserving essential fermentation products. The relative ratio of acetate to propionate (A:P) plays a crucial role in determining the overall energy production derived from rumen fermentation, and it has a direct impact on the partitioning of energy [29]. Moreover, the A:P ratio is linked to methane emissions [30]. An increased A:P ratio has been found to be correlated with elevated levels of methane production, as the readily available H₂ from higher acetate production fuels the hydrogentrophic pathway, leading to increased methane production. This is inconsistent with the findings of Li, Ghimire [31], who observed that the infusion of acetate resulted in an increase in cumulative H_2_ emissions, leading to a higher generation of CH_4_. Also, acetate serves as a precursor for the formation of methane to a lesser extent. In the groups where 10%, 20%, and 30% of silage energy was replaced with a mixture of VFAs, the A:P ratio remained consistent with that of their respective control groups. Nevertheless, silage1/10%VFAs experienced a notable reduction in methane production when compared to the control group. There is a potential for an elevation in the activity of hydrogenotrophic bacteria, which employ hydrogen as a substrate to generate alternative end products, such as acetate or butyrate. It is plausible that these microorganisms could have surpassed the hydrogenotrophic methanogens in competition, resulting in a decline in methane production [32,33]. However, the slightly higher acetate-to-propionate ratio could potentially suggest a rise in other fermentation routes, such as acetogenesis. This process allows them to bypass the conventional methanogenic pathway [28]. This could contribute to the elevated acetate and H_2_ levels and the concurrent reduction in methane emissions. These findings underscore the robustness of the rumen ecosystem and its ability to adapt to dietary changes, which is of utmost importance in rumen function.

In light of the results, it was observed that the concentrate generated methane at a significantly higher rate during the initial 8 h incubation period compared to silage incubation. The elevated levels of methane production observed in concentrate samples could be attributed to their significant levels of readily fermentable starch, sugars, or hemicellulose. These compounds serve as substrates for rumen microorganisms, leading to the formation of gas. The grains included in the concentrate possess a high content of nitrogen-free extract, which might be readily assimilated by rumen microorganisms and serves as a substantial source of substrates for methane generation. The results of the current study align with Bonhomme and technology [34], which indicates that concentrate serves as a favorable substrate for ciliate protozoa, leading to the production of a significant amount of hydrogen and the stimulation of methanogenesis pathways. Furthermore, the substitution of higher energy levels of concentrate (20% and 30%) with VFA mixture led to a greater removal of easily fermentable carbohydrates, such as starch, from the feed. This substitution also resulted in a decrease in methane production when compared to the control groups. However, there were no significant differences observed in total VFA production. Consequently, the supplementation of VFAs in higher energy levels could potentially inhibit methane production without adversely affecting VFA production.

Following the initial 8 h period, it was observed that methane production from the concentrate reached a state of equilibrium, indicating the depletion of easily available sugars and the subsequent initiation of microbial fermentation of less readily degradable carbohydrates. This transition is expected to result in a deceleration of methane production rate. The observed trend of methane production from the silage aligns with the proposed hypothesis. The starting point of methane production in the silage was delayed due to the presence of carbohydrates with slower degradability, as indicated by higher levels of NDF and ADF (Table 1). Consequently, microbial fermentation of these carbohydrates commenced after 32 h. The results corroborate those of Herrera-Saldana, Gomez-Alarcon [35], who found that oat has a comparatively high concentration of soluble carbohydrates, degrades rapidly in two hours, and then rarely breaks down over time because of its complex granule structure. Conversely, a higher crude fiber concentration in the substrate will result in a lower methane generation. According to Lee, Lee [36], there is a negative correlation between the amount of crude fiber in feed ingredients and the in vitro production of methane. Grain methane production falls with increasing crude fiber. In general, the findings of this study indicate that the choice of feed and the carbohydrate content therein may have a notable influence on the rate and pattern of methane production during the ruminal fermentation process of feed compositions supplemented with VFA mixture.

The replacement of a 10% concentrate energy with the VFA mixture led to a decrease in the H_2_ level and an increase in the CH_4_ level, as compared to the control group. This change in conditions resulted in a favorable rise in methane production. Methanogens may have undergone adaptations in response to decreased hydrogen availability, resulting in an increased production of methane to use surplus electrons and sustain a balanced redox state [37,38]. The observed trend did not align with the replacement of greater energy level with the VFA mixture. The experimental group Con3/30%VFAs did not exhibit reduced levels of H_2_, as well as lower amounts of methane, in comparison to their respective control groups. It might be argued that within these treatment groups, the copious VFAs may function as electron acceptors, thereby utilizing a portion of the hydrogen generated. These phenomena can be explained by considering the capacity of VFAs to function as electron sinks, thereby redirecting electrons away from the generation of hydrogen [30,38]. VFAs are known to readily accept electrons through various biochemical reactions. As a consequence, the surplus electrons that would otherwise contribute to hydrogen production may be redirected towards the reduction of VFAs, forming reduced organic compounds [39]. This diversion of electrons from hydrogen production potentially explains the observed reduction in hydrogen levels. Another reason could be the VFA profile. The elevated levels of butyric acid observed in the experimental groups have a complex impact on methane production. Butyrate can act as both a precursor and a competitor in methane synthesis. While it can serve as a precursor for methane, it competes with other VFAs like acetate for hydrogen and electron utilization. Since butyrate generates less methane per unit of hydrogen compared to acetate, higher butyrate levels may redirect more available hydrogen toward butyrate formation, thereby reducing its availability for methane production [36].

The distinct results observed in the silage and concentrate trials can be attributed to the unique nutritional characteristics of these dietary constituents and their interactions with supplemented VFAs. The complex fiber structure of silage posed a greater level of difficulty for microbial fermentation, leading to a decrease in the rate and efficiency of VFA production. On the other hand, the utilization of concentrate, which contains a high amount of easily fermentable carbohydrates, resulted in a more rapid and robust microbial reaction to the supplemented VFAs, ultimately leading to an augmentation in VFA synthesis. Zicarelli, Calabrò [40] conducted an experiment where they incubated six iso-protein diets with varying ratios of forage to concentrate, using rumen fluid. The researchers discovered that organic matter degradability, cumulative gas production, and VFA production was raised as the amount of concentrate in the diet increased. This emphasizes the crucial significance of both the composition of the diet and the inclusion of supplementary VFA mixture in influencing the kinetics of rumen fermentation.

The present study showcases the potential of VFA supplementation to influence methane production and VFA profiles in the ruminal fermentation. The results offer new perspectives on the complex connections among feed composition, microbial activity, VFAs, and gas production. The findings emphasize the potential of VFAs as a valuable supplement to animal feed, which can help reduce greenhouse gas emissions and enhance feed efficiency in ruminant husbandry. The practical implications of these findings are substantial. Integrating VFAs into animal feed formulations presents possibilities for creating novel feeding strategies customized for specific animal categories and production systems. Nevertheless, the economic viability of supplementing VFAs necessitates additional research, encompassing the expenses associated with VFA production as well as the potential economic advantages of decreased methane emissions and enhanced feed efficiency. Examining the potential connections between the production of VFAs and other on-farm activities, such as the generation of biogas, could provide extra economic benefits.

Although the in vitro study showed promising outcomes in decreasing methane production and increasing VFA production by supplementing with a VFA mixture, it is important to recognize the inherent limitations of this experiment. In order to apply these findings to actual feed formulations, it is necessary to conduct thorough in vivo investigations to confirm their effectiveness in real-life situations. Furthermore, conducting thorough research is necessary to develop economically feasible techniques for producing and integrating VFA mixture into animal feed. Accurately measuring the possible decrease in methane emissions in agricultural settings will require a thorough examination of factors such as feed intake, digestibility, and animal performance. Therefore, it is essential for future research to give priority to conducting in vivo trials, performing economic modeling, and optimizing the composition of VFAs to close the divide between laboratory findings and their practical implementation in livestock production.

## 5. Conclusions

In conclusion, this study offers a thorough investigation of rumen fermentation responses to the addition of VFAs to both silage and concentrate. This work elucidates the complex interaction between VFAs, as well as the consequent generation of biogas and VFAs. When 10% of the energy in concentrate was replaced with VFAs, it led to an increase in methane production but a drop in hydrogen levels. Nevertheless, replacing 20% and 30% of concentrate’s energy with VFAs resulted in decreased methane production while preserving the total VFA yield. When considering the energy replacement in silage, replacing 10% of the energy with VFAs led to a reduction in methane emissions and an increase in overall VFA output. In order to make a total mixed ration by silage and concentrate, the optimal condition to reduce methane and increase energy availability is to replace 10% energy of silage and 20% energy of concentrate with a VFA mixture. These discoveries show the potential of adding VFAs as supplements to enhance measures for reducing methane emissions while serving as a valuable energy source for the animal body. These findings provide possibilities for creating novel feed strategies to improve feed efficiency and decrease the negative environmental impacts of ruminant production. Future research should prioritize the optimization of VFA production and the evaluation of the long-term impacts of VFA supplementation on animal health and productivity.

## Figures and Tables

**Table 1:** Chemical composition and energy content of feed and VFA mixture used as a substrate for in vitro incubation.

Characteristics of Feed		Ingredients
	Hay Silage	Concentrate
Dry matter (DM)	428.6 ± 0.3 g/kg	884.9 ± 0.5 g/kg
Moisture	571.4 ± 0.1 g/kg	113.2 ± 0.8 g/kg
Acid detergent fiber	502.4 ± 0.3 g/kg	322.6 ± 0.1 g/kg
Neutral detergent fiber	513.5 ± 0.15 g/kg	398.6 ± 0.3 g/kg
Crude fiber	471.7 ± 0.6 g/kg	293.8 ± 0.4 g/kg
Ash	82.1 ± 0.49 g/kg	51.5 ± 0.4 g/kg
Organic matter	917.9 ± 0.6 g/kg _DM_	948.5 ± 0.4 g/kg _DM_
Gross energy content	18.29 ± 0.2 MJ/kg _DM_	20.17 ± 0.1 MJ/kg _DM_
Starch	ND	250 ± 0.1 g/kg
Protein	113.2± 0.15 g/kg	360.6 ± 0.2 g/kg
**Characteristics of VFA mixture**		
Total VFAs	18.35 g/L	
Acetate	7.28 g/L	
Propionate	1.61 g/L	
Butyrate	9.46 g/L	
Ammonium nitrogen (NH^4+^–N)	1110 mg/L	
Total COD	14 g/L	
Energy content	378.01 J/mL	

**Table 2:** Experimental design of feed and VFAs incubated by rumen media mixture.

Number	Condition	Removed Feed Energy (J)	Replaced Energy with VFAs Mixture (mL)	Set pH
**Silage experiment**				
1	Silage1/10%VFAs	731.6	1.9	7.63
2	Silage 1	-	-	7.63
3	Silage2 /20%VFAs	1463.2	3.8	7.81
4	Silage 2	-	-	7.81
5	Silage3/30%VFAs	2194.8	5.7	7.93
6	Silage 3	-	-	7.93
7	Blank	-	-	7.52
**Concentrate experiment**				
1	Con1/10%VFAs	806.3	2.1	7.42
2	Con1	-	-	7.42
3	Con2 /20%VFAs	1612.6	4.2	7.62
4	Con2	-	-	7.62
5	Con3 /30%VFAs	2418.9	6.3	7.83
6	Con3	-	-	7.83
7	Blank	-	-	7.58

**Table 3:** Comparison of fermentation parameters in concentrate with varying VFA supplementation levels.

Response	Conditions						
	Con1/10%VFAs	Con1	Con2/20%VFAs	Con2	Con3/30% VFAs	Con3	SEM
H_2_ mL	4.74 ^b^	5.85 ^a^	3.57 ^c^	5.21 ^b^	1.62 ^d^	1.99 ^d^	0.08
CH_4_ mL	22.44 ^a^	17.8 ^cd^	14.65 ^c^	18.26 ^b^	13.29 ^c^	14.77 ^c^	0.35
CO_2_ mL	25.52 ^a^	23.12 ^a^	19.49 ^de^	24.69 ^a^	18.01 ^f^	21.53 ^de^	0.42
TVFAs g/L	4.31 ^de^	4.35 ^de^	5.48 ^b^	4.28 ^de^	6.29 ^a^	5.15 ^bc^	0.13
ΔTVFAs g/L	1.56 ^c^	2.79 ^b^	2.85 ^ab^	2.49 ^b^	3.08 ^ab^	3.38 ^a^	0.18
Acetic acid g/L	2.39 ^d^	2.7 ^cd^	3.03 ^abc^	2.69 ^cd^	3.32 ^a^	3.14 ^ab^	0.04
Propionic acid g/L	0.83 ^bcd^	0.96 ^ab^	0.91 ^abc^	0.9 ^abc^	0.96 ^ab^	1.05 ^a^	0.05
Butyric acid g/L	0.68 ^d^	0.5 ^e^	1.12 ^b^	0.51 ^e^	1.49 ^a^	0.53 ^e^	0.04
A:P ratio	2.89 ^c^	2.8 ^c^	3.32 ^abc^	2.99 ^bc^	3.45 ^abc^	2.99 ^bc^	0.08

H_2_: hydrogen; CO_2_: carbon dioxide; CH_4_: methane; TVFAs: total volatile fatty acids; ΔTVFAs: the difference in total VFA production during 72 h concentrate incubation with and without VFA mixture; A:P ratio: acetic acid to propionic acid ratio; SEM: standard error of the mean. Values with different superscript letters in a row are significantly different at the 5% significance level of the Tukey test.

**Table 4:** Comparison of fermentation parameters in silage with varying VFA supplementation levels.

Response	Conditions						
	Silage1/10%VFAs	Silage1	Silage2/20%VFAs	Silage2	Silage3/30%VFAs	Silage3	SEM
H_2_ mL	0.79 ^e^	0.52 ^f^	0.62 ^f^	0.82 ^f^	0.78 ^f^	2.04 ^g^	0.08
CH_4_ mL	9.98 ^f^	11.92 ^de^	8.58 ^gh^	9.97 ^fg^	7.38 ^h^	7.37 ^h^	0.55
CO_2_ mL	21.85 ^bcd^	22.32 ^b^	17.76 ^ef^	23.89 ^bcd^	18.17 ^f^	21.17 ^de^	0.92
TVFAs g/L	4.66 ^cde^	4.26 ^de^	4.78 ^bcd^	4.03 ^e^	5.22 ^bc^	4.76 ^cd^	0.23
ΔTVFAs g/L	1.52 ^c^	0.78 ^cde^	0.18 ^e^	1.17 ^cd^	0.51 ^de^	1.36 ^c^	0.28
Acetic acid g/L	2.84 ^bc^	2.66 ^cd^	2.77 ^bcd^	2.63 ^cd^	2.86 ^bc^	3.05 ^abc^	0.14
Propionic acid g/L	0.77 ^cd^	0.75 ^cd^	0.72 ^d^	0.67 ^d^	0.78 ^dc^	0.83 ^bcd^	0.05
Butyric acid g/L	0.71 ^d^	0.46 ^e^	0.94 ^c^	0.45 ^e^	1.2 ^b^	0.53 ^e^	0.04
A:P ratio	3.69 ^ab^	3.52 ^abc^	3.83 ^a^	3.93 ^a^	3.67 ^ab^	3.68 ^ab^	0.24

H_2_: hydrogen; CO_2_: carbon dioxide; CH_4_: methane; TVFAs: total volatile fatty acids; ΔTVFAs: the difference in total VFA production during 72 h concentrate incubation with and without VFA mixture; A:P ratio: acetic acid to propionic acid ratio; SEM: standard error of the mean. Values with different superscript letters in a row are significantly different at the 5% significance level of the Tukey test.

## Data Availability

All the data results involved in this study have been presented in the article.
